# Role of Virus-Related Chronic Inflammation and Mechanisms of Cancer Immune-Suppression in Pathogenesis and Progression of Hepatocellular Carcinoma

**DOI:** 10.3390/cancers13174387

**Published:** 2021-08-30

**Authors:** Melissa Borgia, Michele Dal Bo, Giuseppe Toffoli

**Affiliations:** Experimental and Clinical Pharmacology Unit, Centro di Riferimento Oncologico di Aviano (CRO), IRCCS, 33081 Aviano, Italy; melissa.borgia@cro.it (M.B.); mdalbo@cro.it (M.D.B.)

**Keywords:** HCC, genetic alterations, tumor microenvironment, pathogenesis, progression, recurrence, anti-viral therapies

## Abstract

**Simple Summary:**

Hepatocellular carcinoma pathogenesis is dependent on a chronic inflammation caused by several factors, including hepatotropic viruses, such as HCV and HBV. This chronic inflammation is established in the context of the immunotolerogenic environment peculiar of the liver, in which the immune system can be stimulated by HCV and HBV viral antigens. This complex interaction can be influenced by direct-acting antiviral drug treatments, capable of (almost totally) rapidly eradicating HCV infection. The influence of anti-viral treatments on HCC pathogenesis and progression remains to be fully clarified.

**Abstract:**

Hepatocellular carcinoma (HCC) can be classified as a prototypical inflammation-driven cancer that generally arises from a background of liver cirrhosis, but that in the presence of nonalcoholic steatohepatitis (NASH), could develop in the absence of fibrosis or cirrhosis. Tumor-promoting inflammation characterizes HCC pathogenesis, with an epidemiology of the chronic liver disease frequently encompassing hepatitis virus B (HBV) or C (HCV). HCC tumor onset and progression is a serial and heterogeneous process in which intrinsic factors, such as genetic mutations and chromosomal instability, are closely associated with an immunosuppressive tumor microenvironment (TME), which may have features associated with the etiopathogenesis and expression of the viral antigens, which favor the evasion of tumor neoantigens to immune surveillance. With the introduction of direct-acting antiviral (DAA) therapies for HCV infection, sustained virological response (SVR) has become very high, although occurrence of HCC and reactivation of HBV in patients with co-infection, who achieved SVR in short term, have been observed in a significant proportion of treated cases. In this review, we discuss the main molecular and TME features that are responsible for HCC pathogenesis and progression. Peculiar functional aspects that could be related to the presence and treatment of HCV/HBV viral infections are also dealt with.

## 1. Introduction

Hepatocellular carcinoma (HCC) is the most common liver cancer, accounting for more than 80% of diagnosed cases among liver cancers. Patients with HCC show poor prognosis, with a 5-year survival rate of 18%. HCC is evaluated as a multifactorial, complex, and sequential disease [1]. Analysis of incidence data shows that death related to HCC is expected to grow to more than 1 million in 2030 [2]. Currently HCC ranks fourth in terms of cancer-related death, and sixth when the number of new cases for a year has been evaluated [3].

Despite its molecular heterogeneity, HCC can be considered as an archetype of cancer, which is based on chronic inflammation caused by infection by hepatotropic viruses, such as hepatitis B (HBV), hepatitis C (HCV) hepatitis D (HDV), liver cirrhosis, metabolic conditions, such as obesity and diabetes, or non-alcoholic liver steatosis, and exposure to environmental/dietary carcinogens, such as aflatoxin b1 [4,5].

Screening, surveillance of patients at risk in combination with vaccinations against HBV and treatment against the HCV, previously based on interferon (IFN), and now based on direct-acting antiviral drugs (DAA), have allowed an improvement in the management and survival of HCC cases, but not in the total eradication of this disease [4,5]. Additional risk factors include the geographic area with the highest incidence in East and Southeast Asia, as well as the Central and West Africa, the male sex (twice as much as females) and a mean age (of HCC diagnosis) of 69, 65, and 62 years in Japan, Europe, and North America, respectively [6].

The pathogenesis of HCC disease was hypothesized to start with the dysfunctionality of cirrhotic or non-cirrhotic hepatocytes caused by the mutations induced by the HBV conjugated to further risk factors, such as aflatoxin B1, and involves an accumulation of genetic variations, including inactivation of tumor suppressor genes, such as TP53, and the activation of proliferation pathways, such as Wnt/FZD/β-catenin and PI3K/Akt/mTOR [5,7,8,9]. On the other hand, in the presence of an excessive liver lipid storage, nonalcoholic fatty liver disease (NAFLD) could arise. In several cases, NAFLD progresses causing hepatic inflammation and liver damage and evolves in the nonalcoholic steatohepatitis (NASH). NASH can cause HCC, in the presence or absence of fibrosis and cirrhosis [10,11].

Tumor onset and progression is a serial and heterogeneous process in which intrinsic factors, such as genetic mutations and chromosomal instability, are closely associated with the presence of an immunosuppressive tumor microenvironment (TME), which favors the evasion of tumor neoantigens to immune surveillance [12,13]. Moreover, the TME may have features associated with the etiopathogenesis and expression of viral antigens that can influence the antitumor response of the immune system [14].

In the last years, the idea of the presence of a highly immunosuppressive environment that participates in the malignant transformation of normal cells, and in tumor onset and progression, has led to an increase in the clinical practice of the use of immune checkpoint inhibitors (ICI), such as anti-PD-1, anti-PD-L1, and anti-CTLA4 immunotherapies, which has been particularly consolidated for melanoma and non-small lung carcinoma treatment. On the other hand, the possible use of ICI for HCC treatment is still uncertain and has to be further evaluated. In this context, the existence of a possible correlation between viral status with the expression of viral antigens and response to drug treatments, in particular immunotherapies, such as ICI, is still to be clearly defined.

The present review describes the main molecular and TME features that are responsible for HCC pathogenesis and progression. Peculiar functional aspects that could be related to the presence and treatment of HCV/HBV viral infections are also dealt with.

## 2. Staging and Clinical Management of HCC

Molecular heterogeneity is closely related to tumor staging with inevitable repercussions in clinical management. Treatment decision making in HCC is based on the Barcelona clinic liver cancer (BCLC), algorithm introduced in 1999, which currently represents the reference methodology used for staging [5,15].

Patients whose diagnosis occurs in the early stages of the disease, defined as BCLC stage 0 or A, depending on the lesions and comorbidities, can undergo radical therapies, such as surgical resection, transplantation, or local ablation, which lengthen the natural survival of the disease by 24 months. The requirements for the access to treatments for the initial stage are different. In fact, patients who are candidates for surgical resection must have preserved liver function with clinically insignificant portal hypertension, ensuring remarkable survival results at 5 years of follow-up (>60%) and post-operative mortality (3%). Surgical resection is generally not able to reduce the percentage of relapses irrespective of the choice of the applied adjuvant therapies [16]. Patients who are not candidates for surgical resection, but who have a single nodule of 5 cm in diameter or three nodules with a maximum of 3 cm each, according to the criteria of Milan, can benefit from transplants with high survival rates with limitations in the presence of tumor invasion macrovascular and extrahepatic diffusion [5,15].

Finally, among the proposed treatments of the initial stage of the pathology, there is local ablation with radiofrequency, in which the high temperature and the consequent tumor necrosis allow smaller clinical complications. In this context, tumors of greater dimensions can be difficult to manage, in case of local ablation with radiofrequency, when compared to surgical resection [17,18].

Intermediate stage (BCLC stage B) patients may benefit from transarterial therapies, such as transarterial chemoembolization (TACE) or selective internal radiation therapy (SIRT) as defined by median survival data [19,20]. These treatments are capable of inducing an embolization of the arteries through beads, with consequent prolonged and slow release of the chemotherapeutic agents and/or through glass or resin microspheres with the radioisotope yttrium-90 (Y90) [19,20].

The use of TACE combined with systemic therapies, such as sorafenib and brivanib, as well as chemotherapeutic agents, such as doxorubicin, fluoropyrimidine, and irinotecan, do not guarantee survival improvement [21,22,23].

Survival improvement is also not guaranteed by established chemotherapeutic agents, such as doxorubicin, fluoropyrimidine, and irinotecan, which are only minimally effective in managing HCC.

However, patients at the advanced stage (BCLC stage C) can benefit from systemic therapies, employing tyrosine kinase inhibitor family molecules, such as sorafenib, regorafenib, lenvatinib, cabozantinib targeting pro-angiogenic, and oncogenic factors, VEGFR1–3, FGFR 1–4, PDGFR alpha, RET, KIT, with the inhibition of multiple oncogenic and angiogenic pathways implicated in tumor progression and metastasis, with a significant benefit of survival. In particular, sorafenib, a small molecule administered orally and approved as a first-line treatment, has demonstrated in a randomized, placebo-controlled phase III trial called SHARP, a median survival of 10.7 months [24]. Patients who showed progression following treatment with sorafenib could benefit from another effective member of the tyrosine kinase family, regorafenib, which provided a 10.6-month overall survival (OS) benefit compared with the placebo. Among the drugs that play a multiple action on factors and pathways that drive angiogenesis, such as VEGFR1–3, PDGFRα, RET, KIT, lenvatinib has been proposed for patients in first line treatment and non resectable HCC, whereas cabozantinib represents a valid option for second-line treatment patients with advanced HCC.

The range of possible treatments for the HCC patient management has been expanded by the use of immunotherapeutic drugs with emerging clinical benefits. These include nivolumab, pembrolizumab, and ramucirumab. The clinical efficacy of nivolumab, an antibody that acts as ICI upon binding to the programmed death-1 receptor (PD-1) and blocking its interaction with PD-L1 and PD-L2 on tumor cells, has been demonstrated through two relevant clinical studies of comparison with sorafenib. The first clinical study is CheckMate 040 [25,26], in which the manageable safety of the drug was also reflected on health status and quality of life, while the tolerability of treatment was investigated through CheckMate 459 [27], where benefits emerge, in terms of OS and objective response rate (ORR), when compared to sorafenib. The second ICI is pembrolizumab, another humanized IgG4 monoclonal antibody that, similar to nivolumab, acts with high affinity, directly inhibiting the binding of PD-1 to its PD-L1 and PD-L2 ligands. Despite encouraging results, in terms of, for example, ORR, as found by data from the KEYNOTE-224 phase II study [28], and the subsequent confirmation study KEYNOTE-240 [29], the clinical efficacy of pembrolizumab is still being investigated. Positive results seem to be provided by an additional monoclonal antibody, ramucirumab, completely humanized for IgG1, whose target is the vascular endothelial growth factor receptor 2 (VEGFR-2).

This binding to VEGFR-2 leads to the inhibition of ligand-induced proliferation and the migration of endothelial cells. In particular, through the controlled placebo study “REACH-2” [30], it has been found that, in a subgroup of patients with HCC having alpha-fetoprotein levels (AFP) of at least 400 ng/mL, the administration of ramucirumab led to an improvement in terms of OS and progression free survival (PFS) [31]. However, further aspects need to be considered for the use of ramucirumab for HCC treatment. In fact, the presence of a cutoff with high levels of AFP restricts the use of therapy to a specific subset of patients with HCC; moreover, the improvement of OS seems to be marginal with a median OS of 1.2 months [31].

Finally the anti-CTLA4 antibody tremelimumab showed relevant results in patients with HCC and coinfection of HCV with a partial response rate of 17.6% and a disease control rate of 76.4% [32]. Recent information presented at ASCO has brought out data from the global Phase II Study 22 trial in which a single initial dose of tremelimumab with added durvalumab every four weeks, showed promising clinical benefits, with median OS of 18.7 months and 24% ORR associated with tolerability, in patients with advanced HCC [33].

## 3. Role of Genetic Alterations in the HCC Pathogenesis and Progression (Intrinsic Factors)

Exploratory analyses of the peculiar mutational landscape of HCC have identified different molecular signatures with the involvement of signaling pathways that drive malignant transformation.

Mutational events in key genes, such as *TERT*, *TP53*, *CTNNB1*, *MYC*, *RB1*, *AXIN1,* and genes involved in chromatin remodeling, such as *ARID1A*, *ARID2*, and *BAP1*, were found to be associated with the malignant transformation process in HCC by TCGA analysis [34]. In addition, inactivations were detected in the genes coding for albumin and the secretion of VLDL, respectively, *ALB* and *APOB*, in a context of increased reactive oxygen species as demonstrated by mutations on *NFE2L2* and *KEAP1*, important in the protection of oxidative stress [34]. This mutational pattern becomes useful to satisfy the energy demand of HCC tumor cells [34].

In an exome sequencing study that analyzed samples of 82 patients with HCC, most of whom underwent surgical resection to treat early stage liver cancer with heterogeneous etiology and absence of dysplastic nodules, 389 somatic mutations were identified as associated with a worse clinical course. There was a relapse in the case of patients with mutations on genes previously identified by TCGA analysis, and in particular, it emerged that loss of *PKD1*, *KMT2D*, and *ARID1A* promoted clonal expansion [35].

Two mutational hotspots upstream of ATG in the *TERT* gene have been found in 60% of cases accompanied by amplification, translocation and viral insertion events. Along with the somatic mutations that occur in this gene, during the beginning and advance of the tumor, in the 30% of HCC cases mutations in the oncosuppressor *TP53* and in the *CTNNB1* genes have been observed, coding for the beta catenin and component of the Wnt pathway, together with the axis inhibition protein1 (*AXIN1*), found in the 10% of cases [35].

Two macro classes defined as proliferative and non-proliferative with a direct implication of mutations in two driver genes, respectively *TP53* and *CTNNB1*, have been proposed.

In the case of the proliferative class the aggressive clinical course is accompanied by the co-presence of HBV infection, a high serum level of AFP and the presence of chromosomal instability with activation of pro-proliferative signaling pathways, such as mTOR and MAPK [5,36]. In these patients, a high presence of *TP53* mutations and *ATM* mutations have been found, conjugated to molecular and pathological aspects, such as the presence of small nodules and *FGF19* amplifications. In addition, the proliferative class has been found to be characterized by a poor cell differentiation accompanied by a micro- and macrovascular invasion with the formation of new arteries and new capillaries fed by the hypoxic environment and epithelium mesenchymal transition [36]. In contrast, the non-proliferative class has been found to be characterized by an increased presence of mutations on the oncogene *CTNNB1*, an etiopathogenesis mainly related to HCV infection, a low macrovascular invasion accompanied by low levels of AFP, maintenance of markers of differentiation and hepatocellular function, and poor cell proliferation [5,36]. As a result, patients belonging to the non-proliferative class were associated with a better clinical course and downregulation of different components of the IL6/JAK/STAT pathway [5,36].

In case of a proliferative status and mainly with concomitant cirrhotic disease, HCC tumorigenesis is characterized by the presence of clonal hepatocytic groups defined as foci and dysplastic nodules to which follows the accumulation of genetic and cytogenetic mutations at the expansion of premalignant lesions [37]. These can be associated with the upregulation of pathways involved in cellular immortalization, cell progression, and permanent regeneration of the fibrotic liver, as well as with the inhibition of apoptosis [34]. In patients with concomitant cirrhosis, the mutation and consequent reactivation of the *TERT* gene is associated with a dysfunction of the hepatocytes with subsequent chromosomal instability, reduced capacity for regeneration and progression of the fibrotic state [38,39,40].

Genomic instability is due to the gain or loss of chromosomal segments, in chromosomes 1q and 8q, 8p, and 22q, respectively, which feed chromosomal numerical abnormalities, supporting phenomena such as aberrant spindle assembly and chromatin cohesion defects. In the early stage of hepatocarcinogenesis the loss of 4q, 8p, 16p, and 17p has been also found [41]. Consequential events occurring during tumor progression from healthy liver to diseased liver were identified and represented in a whole genome study examining 482 microdissections from cirrhotic liver with different pathogenesis (ARLD) and non-alcoholic fatty liver disease (NAFLD) compared to those obtained from healthy liver. In particular, 30 most mutated cancer genes have been identified in samples affected by cirrhosis with simultaneous observation of hepatocytes that accumulate contemporary mutational events in a context of regeneration and toxicity [42]. In contrast to the cluster in which HCC develops as an evolution of liver cirrhosis, a second molecular cluster is represented by patients with hepatic adenomas in which hepatocarcinogenesis may occur. An initially incomplete characterization of pathogenesis of this rare benign tumor has been proposed for women of childbearing age using oral contraceptives [43,44]. Recently, several gene mutations have been identified contributing to cancer pathogenesis and its negative complications, such as bleeding and malignant transformation [45,46,47].

Among the genetic alterations contributing to the malignant transformation of hepatic adenoma, there are the activating mutations in the exon 3 of the *CTNNB1* gene. This *CTNNB1* gene mutation has been associated with the inflammatory subtype of tumor hepatocytes, which is characterized by an over expression of acute phase inflammatory proteins like CRP and serum amyloid A (SAA) and the consequent constitutive activation of the IL6/JAK/STAT pathway. Biallelic inactivating mutations of *HNF1A* have been also found, with repercussions on the synthesis of fatty acids in the liver and their accumulation in tumor hepatocytes [46,48,49].

In the 5–19% of patients with hepatic adenoma, mutations in exons 7 and 8 of the *CTNNB1* gene have been found. These mutations, unlike the mutations activating the exon 3, have been weakly associated with malignant transformation due to the poor activation of the proliferation of Wnt-β catenin signaling pathway [47]. Finally, in the scenario of genetic alterations concerning liver adenoma there are also those responsible for the activation of the sonic hedgehog signaling pathway resulting in overexpression of the key transcriptional factor of the GLI1 signaling pathway and a high risk of bleeding [50]. Table 1 describes the main genetic alterations causing the deregulation of key pathways involved in HCC pathogenesis and proliferation.

## 4. Role of TME (Extrinsic Factor)

Heterogeneity and the worst clinical course of HCC patients are also influenced by the variety and complexity of the TME. To evade immune surveillance, the tumor performs an immunosuppressive process called “immune editing”. This process allows the elimination of some malignant cells, and at the same time, the escape of other cancer cells that promote immune tolerance and tumor progression through, for example, presentation of defective antigens or accumulation of immunosuppressive cell populations [52,53].

The complex analysis of the cell composition observed in the TME revealed the involvement of pro-inflammatory and anti-inflammatory cytokines, extracellular matrix, and subpopulations of immune cells that drive malignant growth and suppress apoptosis [54,55].

Within the subpopulation of immune cells constituting the protection against tumor, a key role is played by regulatory T cells (Tregs), macrophages, cytotoxic CD8+ lymphocytes, and stromal components, such as natural killer cells, Kupffer cells, and dendritic cells [55].

In particular, the balance between Tregs and T cytotoxic constituents of lymphocytes infiltrating the tumor is involved in tumor progression and immune response [56].

In this context, it has been found that pro-inflammatory signals, such as the chemokine axis CCR6-CCL20, IL-10, TGF-β actively participate in the activation of Tregs. There is also a possible role even of long non-coding RNAs [57,58]. Among the latter can be counted the Lnc-EGFR that through a cascading mechanism involving the axis NFAT-P1, important for cell survival or cell death checkpoint for developing T cells, promotes immunosuppression [58].

In addition, the CD4+ CD25 Foxp3+ Treg subgroup can impair the cytotoxic efficacy of CD8+ T cells by interfering with the Caspian cascade and apoptosis, inhibiting the release and production of granzyme A, granzyme B and perforin, or by affecting their activation through the suppression of certain molecules, such as TNF-α and IFN-γ [59,60]. On the contrary, in an advanced stage of HCC, the expression of perforin has been shown to be inversely related to the expression of the *CXCR4* gene whose blocking activates the migration and the consequent capacity of tumor killing [54,61].

Factors, such as hypoxia and the lack of CD4+, also contribute to the limitation of specific responses of associated tumor antigens (TAA) to CD8+ T cells and poor IFN-γ production by the CD8+ T cells in addition to the high expression of a large amount of immunoregulatory molecules in T cells or HCC cells. All of this can be further aggravated by the fibrotic liver condition in which the reduction of the infiltrated CD8+ T cells alters platelet-derived CD44 recognition to perform immunosurveillance. An in-depth investigation has shown that cytotoxicity of CD8+ cells is particularly inhibited in an advanced stage of the disease by the down regulation of CX3CR1, FGFBP2, GNLY, and NKG7 genes [54]. Characterization of the global immune infiltration during the various stages of tumor progression has shown that cytotoxic CD8+ T lymphocytes are present in a higher percentage in early stage patients associated with improved survival [59].

In a current transcriptomic study of immune cells combined with bioinformatics analysis, flow cytometry and immunohistochemistry, effector CD8+ T cells have been shown to have different cell distribution during tumor progression with a reworking of the TME. The upregulation of genes, such as *FOS, JUND*, *JUNB,* and *JUN* responsible for the response to cell stress completes the picture of the altered antitumor response of CD8+ T cells with subsequent cellular reprogramming [54].

Derived myeloid immunosuppressive cells (MDSCs) are also involved in tumor immunity which proliferate uncontrollably accumulating in TME as a result of signals of cytokines, such as G-CSF, GM-CSF, VEGF, MCP-1, and interleukins, such as IL-1β and IL-6 [62].

In mouse models and in patients with fibrotic tissue and HCC, it has been shown how the infiltration of MSCD is associated with poor survival. In particular, this association has been sustained by several mechanisms including poor activation of tumor infiltrating lymphocytes (TIL), inhibition of interferon (IFN)-γ production, and additionally, death of T cells through interaction with the galectin-9 ligand and negative regulation of *TIM3* [63,64].

The sub-populations forming the MDSCs have been clearly characterized by identifying multiple clusters. The most abundant cluster was represented by macrophages with high expression of IL-1β, CXCL10, and CXCL9 followed by macrophages expressed with high expression of coding genes for IFN-γ, TNFAIP3, GBP1, APOBEC3A, and GBP5. In the advanced stage, the presence of macrophages with high CCL18 expression with a metabolic reprogramming has been highlighted as demonstrated by the increase in the metabolism and transport of lipids with a probable support of the hypoxic environment that could involve an overregulation of lipid metabolism during the transition from M1 to M2 states [54].

The most secreted CCL18+ M2 macrophage phenotype is negatively related to survival, large tumor size and TNM stage and at the same time with stimulation of invasion and tumor dissemination and neoangiogenesis [54,65]. Neoangiogenesis causes a dysfunction of the lymphocytes infiltrated tumor following the involvement on the endothelial surface of clustering defective of ICAM-1 and VCAM-1 mediated by the expression of VEGF [66].

It has been demonstrated that VEGF is also capable to induce proliferation of Tregs through the accumulation of MDSCs, the decrease of dendritic cells able to reach a mature state, which in turn contribute to the differentiation and proliferation of Tregs. On the other hand, VEGF is also capable to induce the production of tumor-associated macrophages (TAMs). Angiopoietin-2 (Ang-2) is another proangiogenic factor that has a role in the induction and retention of an immunosuppressive state of the TME. Specifically, the recruitment of monocytes expressing TIE-2 (TEM) can be induced by the expression of Ang-2 in cancer cells. TEM can release IL10 that is associated with the suppression of T cell proliferation, an increase in the ratio of CD4+/CD8+ T cells, and the expansion of Foxp3 + Tregs [66].

Among the innate lymphoid cells (ILCs) directly involved in maintaining the balance between immune defense and tolerance there are the NK cells, which have been found abundant in the human liver. In HCC, the dysfunction of these cells is caused by factors such as severe hypoxia, accumulation of the end products of tumor cell metabolism, inhibitory receptor switches or NK inhibitors (NKRs) as well as by the presence of molecules such as TGF-β, PGE2 or IDO1, and immunosuppressive cytokines such as IL-4, IL-10, and IL-13. These mentioned features have been associated with poor prognosis [67,68]. Phenotypically NK cells can be distinguished into CD56bright and CD56dim, the latter has a predominantly cytotoxic function, as demonstrated by the high expression of granulysin, granzyme B, KIR2DL1 and CX3CR1 [54], while CD56bright CD16low cells regulate the expression of immunomodulatory cytokines such as IFN-γ, TNF-α, IL-1, IL-2, IL-12, IL-15, and IL-18 [69,70]. In HCC patients, there may be a drastic reduction in the number of peripheral NK cells, in particular CD56dim CD16+ NK cells accompanied by a reduced cytotoxic capacity as well as the production of IFN-γ with a close correlation to cancer cell apoptosis and patient survival [71,72]. The efficiency of NK cells can be affected by the action of MDSC cells involved in the inhibition of cytotoxicity and in the production of NK cell cytokines [73], as well as by IDO and PGE2 produced by the HCC cells that downregulate the activation of NK receptors [74].

Finally, 6 subtypes of NK cells have been identified in a recent single cell RNAseq study characterized by different gene expression profiles, showing evidence of the possible role of CD56bright NK cells as precursor cells of Cd56dim NK cells as demonstrated by the different pattern of gene expression [54,75].

A further role in innate immunity is played by dendritic cells (DCs) [76]. In HCC, these cells perform an immunoregulatory action with the involvement of other lymphocytes as happens for example in the alteration of the proliferation of T lymphocytes (through reduced signaling IL-12) and in the promotion of Tregs [77,78]. Several therapies are being tested, such as autologous cell transfer of DCs pulsed with tumor associated peptides, administration of cytokines for TLRs expressed by DCs and administration of tumor associated antigens that are captured by DCs [79,80,81,82,83]. In addition to NK cells and DCs, the components of the tumor stroma include cancer-associated fibroblasts (CAF) crucial players in cancer initiation, growth, and progression, and subsequent remodeling of the TME [84].

The change of the extracellular matrix occurs in a context of chronic liver lesions, caused by the presence of viral agents and oxidative stress. In fact, the tumor secretion of cytokines determines the activation and transformation of quiescent fibroblasts in myofibroblasts resulting in copious production of extracellular matrix proteins that support the development of the fibrotic state [85,86]. Pro-proliferative action by, for example, constitutive activation of JAK1/STAT3 signaling, results in sustained activation of CAFs, combined with an inefficient anticancer response caused by inhibitory action of various cell types including TAMs. The activation of the latter can be supported by STAT3 signaling and is closely related to a poor prognosis with events of tumor invasion and formation of new vessels [87].

Finally, HCC-derived CAFs are involved in the regulation of the viability and functionality of neutrophils through the involvement of the IL6-STAT3-PDL1 signaling pathway and through the induction of chemotaxis of neutrophils as demonstrated by a study that deepened the effects and role of HCC-derived CAFs on the neutrophils [88]. Table 2 summarizes the main functions of immune system-related cells in the modulation of the HCC TME.

## 5. Role of HBV, HCV Infection in the Pathogenesis of HCC (Early Stages)

The liver is an immunological and tolerogenic organ, critical hub for numerous physiological processes, which, if necessary, can activate the immune response to defend against external attacks as demonstrated by the rich resident lymphocytic population that allows to implement a first line of defense against invading pathogens, modulation of liver injury, and recruitment of circulating lymphocytes [55,89,90]. Moreover, among the many functions, a specific mention should be made of the metabolic ones, such as storage of glycogen in the homeostasis of lipids and cholesterol, metabolism of proteins and amino acids, as well as of the filter functions for exogenous substances and the support of the immune system [89].

**Figure 1:**
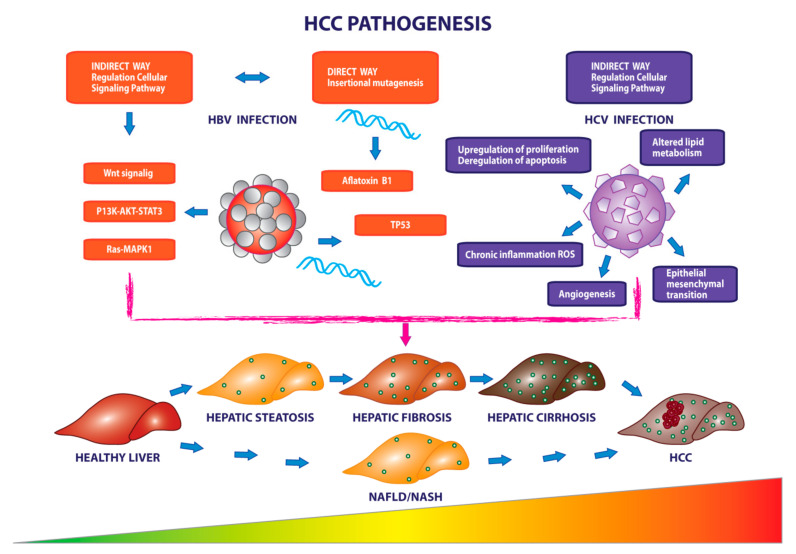
Involvement of viral infections in the pathogenesis and progression of HCC. The pathogenesis of HCC is closely related to the presence of viral infections from hepatitis B and hepatitis C. Viral agents support the onset and progression of cancer through consequential events involving genetic alterations, increased reactive oxygen species and angiogenesis occurring during tumor progression from healthy liver to diseased liver.

Hepatotropic viral infections represent a significant risk factor responsible for the development of HCC in 54% of HBV patients [91,92,93] and in 10–25% of HCV patients worldwide. Moreover, in the 76% of HCC cases the tumor pathogenesis is associated with viral infections. On the other hand, the 24% of HCC cases show a tumor pathogenesis not associated with viral infections (Figure 1) [94].

The study of the etiopathological mechanism triggered by the concomitant presence of the virus has shown the existence of two types of action, according to which the hepatitis B virus may induce carcinogenesis and immune imbalance [95]. The direct way of action is through the integration of the virus in the host genome, causing hotspots of mutations, for example in the promoter of the *TERT* gene. An indirect mechanism is through alterations in the gene expression of specific hepatic microRNAs and in the secretion of oncogenic proteins, such as non-histones X protein (Hbx), which promote replication, viral invasiveness, and subsequent carcinogenicity by regulating the PI3K-AKT-STAT3, Wnt-FZD-β catenin, Ras-MAPK1 signaling pathways [9,96,97]. Regardless of the mechanism, virus-induced chronic liver damage involves necroinflammation, and an increased rate of proliferation and fibrogenesis through immune attack [97,98]. In fact, the presence of acute HBV infection leads to greater and abnormal sensitivity of hepatocytes to the action of death ligands such as TNF and TRAIL, key factors in the regulation of the immune system and apoptosis, and Fas ligand, a member of TNF and mediator of apoptosis of liver-infiltrating lymphocytes [99,100]. Given the key role of TNF in promoting hepatocytic apoptosis, TNF antagonists are effective therapies in blocking viral replication but in patients with active infection and in the presence of detectable serum HBV surface antigen (Hbsag) this treatment may cause reactivation of the HBV virus. The pharmacological action of TNF antagonists is neutralized by the expression of cellular inhibitors of apoptosis proteins (CIAPs), that promote cell survival by supporting viral replication, although their deactivation, through the second mitochondrial-derived caspase activator (SMAC) results in the virus arrest in infected livers as demonstrated in mouse models [101,102,103].

The direct action to the dysregulation of the signaling pathways involved such as PI3K-AKT-STAT3, Wnt-FZD-β catenin, Ras-MAPK1, and the concomitant involvement of SMAC could lead to an ambivalent advantage targeted to cancer cells and to the arrest of viral replication [101]. The cellular mechanisms underlying carcinogenesis caused and controlled by the presence of HBV entail a reproductive advantage to the virus that is reflected, for example, on the progression of the cell cycle [104].

In fact, the intranuclear accumulation of p21CIP1, member of the inhibitor kinase family, in the G2 phase was proposed to activate extracellular signal-regulated kinases (ERKs). Activation of ERK could cause the blocking of the cell cycle in the G2 phase. This, in turn, involves the Wnt-β catenin signaling pathway in the chromatin folding mediated by the Smc5/6 complex and in the transcriptional activity of covalently closed circular DNA (cccDNA) [105,106]. The role of p21CIP1 in the pathogenesis of HCC is also exerted via its involvement in the regulation of immune cell responses. In particular, it has been demonstrated that in HCC *NCOA5^+/−^* mouse model there is an altered expression of several genes including *p21CIP1* that has been associated with the presence of pro-inflammatory cytokines, expression of activated T cells, TRM cells, MDSC, and M2 macrophages. Moreover, CD8 + T cell populations of HCC *NCOA5^+/−^* mice were characterized by the expression of T cell exhaustion signatures. Of note, aberrant p21CIP1 expression and immune-related associated characteristics can be reverted by treatment with metformin. Of note, expression of p21CIP1 in HCC tissues has been demonstrated to be an independent good prognosis factor of survival [107,108].

The cytopathic changes in the context of liver structure induced by the accumulation of HBV viral proteins, even in the absence of active viral replication, have been clearly described in transgenic mouse models. In these models, the transcription of the sequence coding the viral involucre has been shown to involve the synthesis of Hbsag particles that caused their storage in the endoplasmic reticulum with the growth of the same and at the same time a remodeling of the hepatocytes [109]. As a result of morphological changes, hepatocytes acquire characteristics of “Ground-Glass” cells, such as the presence of granular and glassy eosinophilic cytoplasm [109,110]. The intracellular concentration of the envelope polypeptide is closely related to coagulative necrosis of these cells, which is accompanied by focal hepatocellular degeneration, lobular macrophage inflammation, and increased serum transaminase activity [109]. All of the above mentioned pathological changes could be associated with the fact that the accumulation of the viral products in affected cells can occur if the production of the viral products is beyond the secretion capacity of the host cell or the secretion efficiency is restricted by the cellular factors. Moreover, the accumulation of viral products can cause a plethora of significant hepatocytic damages in a similar manner to the excessive accumulation of fats or bile acids in the liver cells responsible for liver injury including fatty liver disease or cholestasis [111], causing the histopathological changes peculiar of hepatitis B. This is in keeping with in vitro results demonstrating that the stable expression of small HBV surface protein in HepG2 and Huh7 cells is capable to increase the susceptibility of these cells to apoptosis [109]. Liver resident memory T (TRM) cells exert a key role in the promotion of an antiviral response in the presence of a viral infection, in particular when this infection becomes a chronic infection. Specifically, CD8 + TRM cells are involved in the control of viral replication and in the maintenance of viral protection in long periods. They express high levels of IL2 and perforin, as well as they secret high levels of pro-inflammatory cytokines. It has been demonstrated that, in mouse models with HCV re-infection, the depletion of liver CD8 + TRM cells has been associated with prolonged virus persistence and an absence of effective liver clearance. On the other hand, virus eradication has been reached in the same models when liver CD8 + TRM cells have been recovered. The role of liver TRM cells has been also investigated in the context of HBV infection. In this case, a specific TRM cell subpopulation is selected. In particular, there is an enrichment of TRM cells in HBV patients who enriched viral control in comparison with healthy cases also in presence of the same total T cell frequencies in the liver. Of note, in several cases, virus-specific CD8+ TRM cells can be found in spontaneously recovered HBV patients, thus highlighting the key role of this cell population in the long-term viral control [112,113,114,115].

During the active state of chronic HBV infection there is the inhibition of immune-related mechanisms capable to control viral replication that is conjugated to a damage of the energy and metabolic cellular apparatus at several levels [95].

In the first case, there is a dysfunction of HBV-specific CD8+ TIM-3+ T cells that become unable to respond effectively to the presence of the virus with the production of cytokines, as demonstrated by the deletion of antigen- specific targeting and their limited proliferation and, conversely, with high levels of expression of inhibitory receptors such as CTLA-4, PD-1, and TIM-3; thus, causing the subsequent progression of the tumor [116,117,118]. This mechanism was further demonstrated to be supported by an additional immunosuppressive profile in CD8+ TRM cells with higher expression levels of PD-1 in the tumor tissue of HBV-related HCC [119]. The depletion of CD8+ TRM cells is also closely related to the impairment of energy and metabolic mechanisms that, in a context of increased reactive oxygen species, involve a downregulation of mitochondrial transport mechanisms with further sub-regulation of transcription and translation of mitochondrial DNA, damage of the apparatus useful for DNA repair, alteration of glucose metabolism with consequences in liver T cell function [120]. Residual populations of specific antigen T cells have been found in patients with chronic hepatitis B in keeping with the hypothesis of a viral contribution in maintaining the highly inflammatory environment and tumor predisposition of the liver [121]. In addition, the use of mouse models has allowed to identify the central role in the development and maintenance of the inflammatory environment of CD8+ TRM cells that secret IFN-γ when activated by the anti-CD137 monoclonal antibody. These cells are involved in the recruitment of liver macrophages that in turn secrete factors such as TNF-α, IL-6, and MCP-1 [122]. Anti-viral and antitumor immune responses can be also regulated by CD4+ T cells by the production of cytokines capable to activate CD8+ T cells and B cells. In this context, it is noteworthy that circulating and liver-infiltrating CD4+ T cells have been found to be increased in the early stage of HCC, in particular with significantly higher levels than those of chronic hepatitis B patients [123]. This seems to indicate that not chronic HBV infection only should be considered as the main responsible for the observed increase in CD4+ T cells in HBV-related HCC. The decrease of both CD4+ T cells number and activity occurring in the progressive stages of HCC seems to be linked with the increment in the specific Treg cell population. Moreover, the progressive deficit in CD4+ T cells has been found to be associated with the high recurrence and poor survival of HCC patients [123]. The suppressive function of Tregs has been found to be exerted via cell to cell contact as well as through the secretion of cytokines such as IL-2, IL-10, TGF-β, and IL-35. Of note, an enrichment of the Tregs number has been found in HBV-related HCC patients. This enriched number was also associated with a higher expression of PD-1, which seems to be involved in the establishment of the more immunosuppressive and exhausted microenvironment peculiar of HBV-related HCC when compared to the non-virus-related HCC [119]. The reduction of the function of CD8+ T cells has also been associated with the increased number of Tregs in HBV-related HCC patients. This is in keeping with the inhibited proliferation and activation of CD8+ T cells as well as with the attenuated cytotoxicity of CD8+ T cells due to a decrease in the production of granzymes A/B and perforin [59]. It has been also reported that the persistent presence of HBV seems to be associated with elevated TGF-β levels, which in turn have been found to suppress miR-34a expression and to enhance CCL22 expression, thus recruiting Tregs in the liver tissue. Moreover, the development of portal vein tumor thrombus in HCC patients seems to be associated with the number of Tregs involved in the immune escape of HBV-positive HCC [124]. The increment in the number of Tregs has been involved not only in the suppression of HBV antigen-specific immune responses, but also in the suppression of HCC tumor antigen-specific immune responses [125]. Further, although the frequency of circulating CD4+ CD25+ CD127− Tregs has been found to be much lower in HCC patients than in healthy donors and patients of chronic HBV infection, HCC resection by surgery has been found to be associated with a significant increase in the frequency of circulating CD4+ CD25+ CD127− Tregs in HCC patients, that seems to be correlated with tumor aggressiveness [126]. Thus, a targeted therapy capable to reduce Tregs could be efficient for the treatment of HCC patients [127].

Similar to HBV, HCV has a wide global diffusion with greater prevalence in industrialized countries [98], but unlike HBV, this RNA viral agent, acts indirectly on the host genome and chronicizes in 70–80% of cases. In fact, viral replication does not occur by integration into the genome but insidiously through the synthesis of nonstructural proteins that support viral proliferation in hepatocytes by altering the MAPK cellular signaling pathway [98,128]. Liver homeostasis is altered by the presence of HCV that through a mechanism of proliferation of hepatocytes allows the blocking of apoptosis, cell shrinkage, and fragmentation of the cell nucleus in liver cells, and the simultaneous viral replication. Moreover, the HCV allows the simultaneous modification of the balance in the deposition of the proteins of extracellular matrix, in response to chronic liver damage, causing the onset of liver deterioration [129,130]. Some evidence has been demonstrated about the pathogenic mechanisms related to viral infection. It has been found that the deposition of proteins of the extracellular matrix, such as laminin, collagen, fibronectin, is as key mediator of the activation of hepatic stellate cells (HSC) that, as a result of an upstream mechanism involving modulation of the metabolic pathways induced by the action of viral proteins, and the consequent antiviral immune response, are transformed into proliferative and contractile myofibroblasts [131]. The importance of genetic variation over environmental issues has been emphasized by the fact that the final response to infection with the induction of pathogenetic mechanisms is not similar among patients with several other factors such as age, duration of infection, insulin resistance (IR), steatosis, and so on, also closely associated with the final outcome of the disease [130]. The HCV viral proteins involved in the activation of HSC appear to be three mature structural proteins (core, E1, E2, and p7) and seven non-structural proteins (NS2, NS3, NS4A, NS4B, NS5A, and NS5B), which through different mechanisms regulate cell proliferation, apoptosis, innate immunity boost, and the inflammatory state supporting liver fibrogenesis [132,133]. Growth factors, inflammatory cytokines and chemokines appear to make a major contribution to the activation of HSC and subsequent transformation into myofibroblasts driving the fibrogenetic process. In fact, in the viral context, a positive regulation of immune responses emerges with the combined action of growth factors, such as PDGF, EGF, VEGF, key mediators of mitogenic, and angiogenetic effects and fibrogenic cytokines that promote the production of ECM [134,135]. In addition, during the activation of HSC, chemokine sharing in fibrogenetic pathways was found through involving chemotactic processes through CXCL5 [136], CXCL9, which promote fibrogenesis and amplify the inflammatory response with a possible role of neurochemical and neurotrophic factors [137].

In response to the viral agent of hepatitis C the immune system implements an immunological response by activating tumor necrosis factors that result in a cyclic pattern of cell damage, apoptosis and regeneration combined with reactive oxygen species that feed genomic instability and carcinogenesis [98]. To have the possibility to treat and eliminate the viral infection, cause of the chronically inflamed environment, means to act also on the possible tumor progression in patients with hepatitis C [138]. Moreover, it should be remembered that in cirrhotic and HCV patients the risk of incurring hepatocarcinogenesis persists, for a period still to be verified, even if the infection is inactivated [139]. Closely related to HCV infection, phenomena of ductal metaplasia have been found, which could contribute to carcinogenesis by epigenetic reprogramming due to the transdifferentiation of mature hepatocytes in bipotential oval cells [140].

However, specialized pericentral liver cells, capable of self-renewal in the uninjured liver under the influence of endothelial Wnt signaling could represent other sources of tumor cell precursors. These cells are diploid and are characterized by the expression of the early liver progenitor marker TBX347. Other possible precursor cells are the hybrid periportal cells that are characterized by the expression of hepatocyte markers, along with low expression levels of SOX9 and of several bile duct genes. These cells are able to repopulate the healthy and diseased liver mass. The real contribution of these cell populations in tumorigenesis remain still to be clarified in particular if they are bona fide stem or progenitor cells or are distinct hepatocyte subpopulations [140].

## 6. Antiviral Therapies and the Role of HBV/HCV Infections in the Modulation of the Anticancer Immune System Response

**Figure 2:**
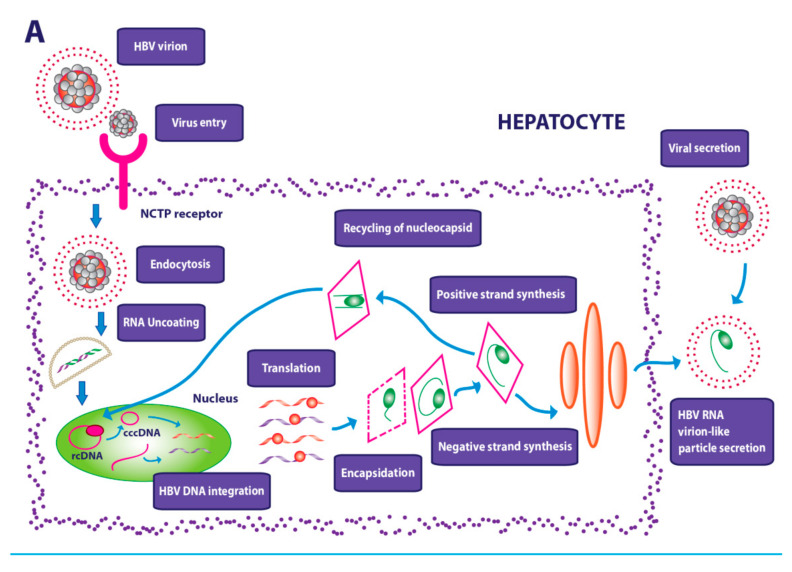

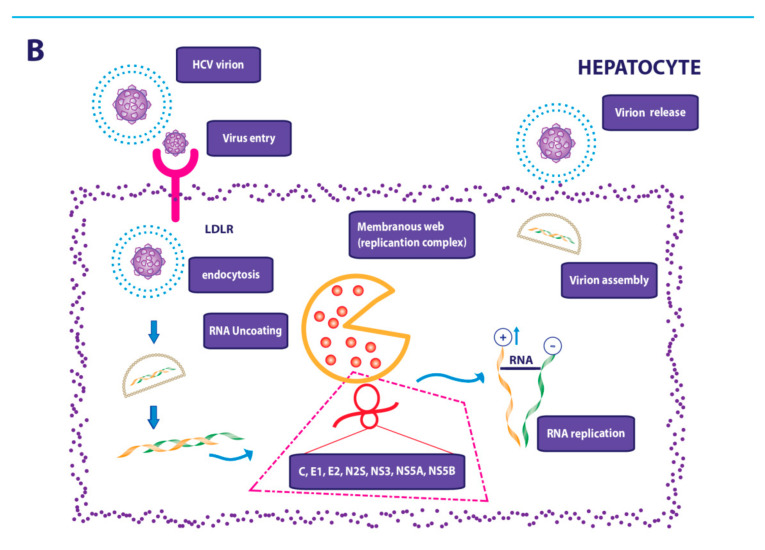
Viral life cycles. (**A**) Hepatitis (**B**) virus life cycle: the virus enters through the link with Na^+^ taurocholate cotransporting polypeptide (NTCP), follows the endocytosis of the viral agent and the transport of nucleocapsid into the nucleus in which the relaxed circular DNA genome (rcDNA) is converted into covalently closed circular DNA (cccDNA). This process allows the transcription of the viral mRNA with the subsequent encapsulation of the viral protein core and the production of the double-stranded linear DNA. Through the endoplasmic reticulum, viral particles are assembled and subsequently secreted. (**B**) Hepatitis C virus life cycle: hepatitis C virus entry is mediated by the low density lipoprotein receptor (LDLR) with subsequent release of the viral genome that undergoes replication and transcription with production of a single polyprotein chain that allows the synthesis of viral proteins. They follow the assembly and release of viral particles.

The ability of HBV and HCV viruses to establish a stable infection makes it difficult to eradicate them (Figure 2A,B).

As a result, the new drug therapies have been directed to the intrahepatic reduction of cccDNA and the consequent reduction of the risk of new infections demonstrating a greater effectiveness in the combination of inhibitors of the entrance of the virus, as myrcludex B, and simultaneous inhibition of viral replication by the use of IFN-α [141]. Moreover, the use of small molecules that inhibit the entry of the virus, such as ezetimibe [142] and cyclosporine [143] derivatives, to which are added monoclonal antibodies against Hb surface antigen epitopes capable of interfering with viral circulation have demonstrated a significant efficacy although they are still under study.

The strategy of the inhibition of the formation of cccDNA is at the base of further therapies that preview the block of the enzymes useful for its formation by means of nuclease zinc fingers, activators of effector nucleases transcription, and CRISPR-Cas9, the use of which has revealed remarkably positive data [144].

In addition, viral chronic conditions are supported by high antigenic load. Therefore, transcriptional and post-transcriptional control by regulating epigenetic mechanisms such as acetylation and methylation could affect cccDNA transcription as demonstrated by several studies [145,146]. In this regard, the C646, a small selective molecule inhibitor of histone acetyltransferase CBP and p300, has been shown to be effective in inhibiting the transcription of HBV from cccDNA [146]. Post-transcriptional control therapeutic strategies include the use of RNA interference, antisense oligonucleotides (ASOs), and ribonucleic acid enzymes (ribozymes) with data potentially emerging from ongoing clinical studies [144]. Therapies, such as core protein allosteric modulators (CpAMs) and capsid assembly modulators, are able to reduce the release of infectious viral particles and block the nuclear transport of nucleocapsids [144]. Finally, immune mechanisms are the basis of therapies directed to modulate innate and adaptive immunity. In the first case, the use of pharmacological therapies may activate the immune response in myeloid cells, natural killer (NK), and invariant T cells associated with the mucosa (MAIT), while in the case of adaptive immunity, it is possible the use of antibodies that can block HBV infection or directly induce cell lysis through the activation of antibody-dependent cellular cytotoxicity (ADCC) or complement dependent cytotoxicity (CDC) [147]. Cytokines, such as TNF, IFN-α, IFN-γ, and IL-1β, or pattern recognition receptor agonists, can involve both innate and adaptive immunity mechanisms [147]. The main phase III clinical trials investigating novel drugs for HBV treatment are reported in Table 3.

Patients with HCV infection can be treated with pegylated INF-α therapy and ribaravin therapy with a different treatment response rate based on the viral genotype [148]. In this combination the antiproliferative and antiviral action of the INF is associated to the multiple molecular mechanisms of the ribaravin causing the direct inhibition of the viral replication or greater frequency of mutations interfering with the replication mechanism, competitive inhibition for de novo synthesis of guanine nucleotides, or immunomodulation through the involvement of helper T cells (Th1) [149,150]. In recent years, clinical management for the treatment of HCV has been expanded by the advent of direct antiviral agents divided into three macrocategories, depending on the molecular target. The first category includes protease NS3 inhibitors (...-previr), such as glecaprevir, grazoprevir; drugs, such as daclatasvir, elbasvir, ledipasvir, ombitasvir, belonging to the class of the NS5A serine protease inhibitors; and finally, the inhibitors of RNA polymerase dependent NS5B (NS5B Rdrp) (...-buvir) subdivided in nucleoside polymerase inhibitors or non-nucleoside polymerase inhibitors [151]. The main phase III clinical trials investigating novel drugs for HCV treatment are reported in Table 4.

In-depth knowledge of immune subset landscape in HCC cancer cells related to HBV/HCV is outlined through transcriptome sequencing on myeloid cells, NK cells, and lymphocytes with a significant impact on the possible use of immunotherapy on clinical outcomes [54]. In a recent meta-analysis conducted by using RNA-seq data from TCGA analysis, the repercussions of viral etiology, and TME were examined by comparing viral infected patients against non-infected patients. The estimated clinical endpoint was represented by objective response rates defined as the number of responders to ICI, such as PD-1 and PD-L1, divided by the total number of treated patients [14]. In particular, the subject of this meta-analysis consists of six clinical trials used with information on the state of response stratified for the presence or co-presence of infection with HBV, HCV compared to non-virally infected. The response rate obtained did not show differences between viral etiology and the response to drug therapy with ICI. In addition, the analysis of the effect of viral etiology on tumor infiltrating lymphocytes by studying the expression of key markers of CD8+ T cells, CD4+ T cells, CD20+ cells, or CD68 macrophages again demonstrated the absence of correlation between viral pathology and biomarkers. The above mentioned analysis has been continued by two further tests focusing on the possible correlation with tumor mutational burden (TMB) and the diversity of T-cell repertoires (TCR) and B cell repertoires (BCR). By performing it, no significant effects of the viral infection have been found [14]. Until recently, the clinical management of the HCV was entrusted to the use of injectable IFN, effective drug therapy although with serious adverse reactions, replaced after a decade by direct action antiviral agents (DAAs) with clearly safety profiles and positive efficacy with an effectiveness of more than 90% in almost all patients, and with positive feedback, even in cases of co-infection with HIV. In any case, it is essential to consider possible co-morbidities that may limit the use of treatment in certain special patient populations [139].

Recently, conflicting data regarding the risk of hepatocarcinogenesis or a more widespread recurrence of HCC after treatment with DAA therapy have required further investigation.

In fact, unexpectedly, it has been showed an increased risk of hepatocarcinogenesis or more diffuse HCC recurrence after treatment with DAA therapy raising several doubts about the optimal timing of HCV treatment in patients with HCC. In particular, in patients with HCC that underwent an anticancer treatment, a successive treatment with DAA after this anti-cancer treatment was associated with a high recurrence rate compared to the incidence already known in patients with successfully treated HCC [152]. Specifically, the patients included in this study had a HCV infection and they were previously treated for HCC with the achievement of a complete response and the lacking of “non-characterized nodules” at the time they underwent a successive anti-HCV treatment with oral DAAs [152]. In post-ablation patient cohort for small HCC the actuarial probability of recurrence was 2.45% (4/163) to 4 months and 27.6% (45/163) to 12 months while the rate of recurrence to 4 months in high- to low-risk patients who had undergone surgical resection was 13.5% and 3.8%, respectively [152]. One possible explanation for this correlation could be that the immune system initially activated by the viral infection, following treatment against HCV could reduce its efficient immunosurveillance resulting in an increased risk of developing HCC [139]. The study showed a close correlation between DAA treatment and the risk of recurrence with an immune environment prone to suppression and cancer proliferation in a context of existing liver damage with complex dissemination mechanisms [152].

A further prospective study of 2018, analyzes data from 55 patients with HCV and advanced liver disease treated with various DAA regimens reporting the risk of developing HCC following antiviral vs. the absence of DAA regimen. Evidence shows similarity or reduction in the risk of hepatocarcinogenesis during the first year of administration of DAAs [153]. In this study, in the majority of the cases, HCC developed at the beginning of the anti-viral therapy, with a frequent association with an unsuccessful treatment. A frequent HCC development in patients in which anti-viral therapy did not achieve SVR was also reported in other studies. In this context, the proposed hypothesis is that HCC cells serve as a viral reservoir for HCV less permissive to DAAs, with the consequent relapse of HCV infection after antiviral therapy. On these bases, patients who did not reach SVR and developed HCC could already have microscopic, undetectable foci of HCC before initiation of DAAs. In particular, it should not be excluded a possible natural progression of the disease given the use of imaging tests prior to the start of antiviral therapy to check for the presence of HCC at baseline. Further elucidations could be provided by imaging tests during and after antiviral therapy at a diversified rate to check for existing comorbidity [153].

In this regard, the risk of developing HCC, in patients with HCV and advanced liver disease in treatment with DAAs, could be related to the co-presence of further pathologies such as HBV and diabetes rather than to the use of antivirals. This risk increases in patients not responsive to antiviral treatment and decreases progressively over time in patients with SVR [153].

In a French multicentric study, adult patients with exclusive chronic HCV infection included were divided into three different cohorts based on pathogenesis or the presence or absence of cirrhosis and fibrotic status not classified with a median follow-up period of 33.4 months. The aim of the study was to investigate the incidence of death, neoplasm, and decomposed cirrhosis in patients treated with DAAs compared to the control group [154]. Preliminary analyses associated the risk of HCC and cirrhosis with the pharmacological treatment administered but following statistical adjustment for variables, such as age, sex, geographical origin, and the presence of possible comorbidities, such as diabetes or high blood pressure, the DAA treatments have been found to be against tendencies with a decrease in all causes of mortality, decomposed carcinoma and cirrhosis related to treatment with DAAs as confirmed by further statistical analysis. In addition, as a result of the adjustment, the overall percentage of patients with SVR was 92% [154]. The achievement of SVR was associated with the decrease of all causes of mortality while the failure of reaching a SVR has been related to the increased risk of HCC insurgence, as demonstrated by multivariate analyses. In the subgroup of patients without cirrhosis or in the presence of an unclassified fibrotic state, the virological response was 96%. Although with limitations that would probably not affect the results achieved, the study showed a lower risk of non-liver-related mortality in patients with DAA when compared to the control group in particular in patients with a prolonged virological response. However, the observational nature of the study requires further investigation to clarify the plausible mechanisms identified in this study, such as the fact that the action of drug treatment reduces liver damage and inflammation, resulting in positive effects on liver complications [154].

A further study supports the conclusions just reported by a retrospective analysis in which a cohort of HCV patients treated with DAA were compared to patients not treated with DAA after reaching a complete resection response, local ablation, TACE, or radiation therapy [155]. The criteria for excluding from the study were interferon or DAA-based therapies prior to the full HCC response or the finding of unknown carcinoma responses. The primary outcome was death caused by liver problems, related to HCC, unrelated to HCC or unknown. Further analysis has been carried out by stratifying the patients for the tumor load, treatment that involves the complete response and recurrence from HCC. Data on the association between DAAs and overall survival showed a higher median time from neoplasm insurgence to death in treated patients when compared to patients not treated with DAA with rates of 25.7 months vs. 11.5 months respectively [155].

Additional investigation showed that DAA therapy was associated with a reduced mortality rate although statically not significant. Analysis of the subgroups showed an improvement in survival in patients with cirrhosis with Child-Pugh A or B classification, in the reduction of mortality in patients undergoing ablation or resection when compared to patients treated with TACE and a benefit found further in patients with significant tumor burden. However, the beneficial effects resulting from the use of direct antiviral agents were higher in non-recurring patients when compared to relapsing patients. (HR, 0.09; IC 95%, 0.02–0.29 vs. HR, 0.86; IC 95%, 0.49–1.52).

This study fits into the scientific controversy over the use of DAA therapy in patients with HCC history supporting the thesis of the aforementioned French study by demonstrating that DAA therapy can also reduce mortality in patients with a history of HCC with statistical limitations in the analysis of subgroups [155].

Nevertheless, the remaining doubts about the appropriate timing to consider for the proper administration of therapy with DAA in relation with the presence or insurgence of HCC were discussed by the American Gastroenterological Association (AGA) in 2019 through a useful update to clinical practice, with the drafting of recommendations regarding the use of DAAs and the appropriate timing of therapy in supervised HCC patients [156]. In particular, the first recommendation concerns the potential hepatocarcinogenic effect in patients treated with DAA, by which a reduction in the incidence of HCC in the treatment group is confirmed when compared to the control group treated with IFN. The risk of developing HCC was greater in patients treated with DAA but who had not reached SVR. The latter associated with the reduction of the relative risk of HCC was similar in groups of cirrhotic and non-cirrhotic patients [156]. It is clarified that attention should be paid to maintaining active surveillance of HCC in patients with SVR induced by DAAs. In particular, the importance of surveillance is found in those with concomitant cirrhosis where the annual risk of HCC varies from 1.8% to 2.5% increasing in the advanced stages of cirrhosis. Supporting data emerge from a study, with 2140 patients treated with DAA with HCV infection and cirrhosis, in which hepatocarcinogenicity was found in 78 patients under regular surveillance prior to antiviral therapy. In addition, the presence of advanced liver fibrosis was related to a high risk of developing HCC as opposed to the absence of cirrhosis that was associated with a low risk of hepatocarcinogenesis. In conclusion, surveillance, to be carried out before antiviral drug therapy, remains essential, in particular in the case of cirrhotic or advanced fibrosis patients together with those who progress in cirrhotic or fibrotic states at the time of SVR. In this case, the AGA recommends ultrasound and assay of alpha-fetoprotein (AFP) in an interval of 6 months [156]. In the case of active HCC, DAA therapy is associated with improved liver dysfunction and additional pharmacological possibilities however with a statistically significant decrease in SVR associated with cancer-related mortality. In fact, as several studies have shown, patients with active HCC treated with DAA have achieved a lower SVR as demonstrated by multi-variable analyses where the presence of the tumor is associated with a lower probability of SVR when compared to patients without HCC (adjusted OR, 0.59; 95% CI, 0.36–1.0). Therefore, treatment of the viral infection should take place before possible carcinogenesis conversely, in patients with active HCC it would be indicated to wait for a complete response to anticancer therapy before antiviral therapy thus allowing benefits in the SVR by evaluating individually the different cases and various variables as to the tumor load and the degree of liver dysfunction [156]. On the contrary, in patients where the full response to HCC was found to be reached, elongating by 4–6 months the interval of time between this and the therapy with DAAs involves a greater possibility to carry out diagnostic investigations allowing a clinical confirmation of the response for the HCC together with the possibility of immune surveillance of tumoral clones. However, limitations in studies, such as heterogeneity between patient cohorts or between treatments used, time lag between carcinoma and antiviral treatment, and post-treatment surveillance with DAA should be taken into account. Due to the risk of recurrence of HCC in patients with a history of HCC and SVR induced by DAA antiviral agents, surveillance should be continued by carrying out diagnostic investigations with intervals of 3–6 months [156].

Prior to the use of DAA, no similar data were found during the treatment with INF since the kinetics of viral suppression and related inflammation are clearly different between the two drug therapies. In fact, in the case of DAA, the elimination of the viral agent occurs quickly in contrast to the regime with INF [139]. The use of INF based therapy could have brought to the cancer some immune advantages seen the action of promotion of the antitumor immunity given by the greater effectiveness on the disseminated tumor cells and on the block of their proliferation to which are conjugated the inhibition cell cycle phases, the regulation of immune cells and angiogenesis [68,104].

The relationship between treatment with DAA and the host immune status has been recently highlighted by results showing that the innate immunity could be reconstituted by DAA therapy and that HCV clearance achieved by sofosbuvir and ribavirin treatment can be associated with the hepatic downregulation of type II and III IFNs, their receptors, and INF stimulated genes. Moreover, recent data regarding hepatitis B virus reactivation during DAA therapy support the concept of the derangement of immune surveillance [152].

In a recent clinical study emerges an epigenetic signature showing a link between the development of HCC following SVR in patients treated with DAA and the maintenance of risk for treated patients [157]. If epigenetic changes occurred following infection, the persistent HCV-specific gene expression pattern is maintained also in absence of the virus with the persistence of its oncogenic effects on the host cells. This persistent effect may be linked to HCC progression. In this context it has been suggested that epigenetic inhibitors could be useful to revert this HCV-induced epigenetic signature as well as the EGFR inhibitor erlotinib, for the involvement of the EGFR in the induction of the epigenetic changes following the infection. In contrast to DAA treatment, the INF treatment seems to not induce a similar persistence of epigenetic signature, in keeping with the hypothesis that HCC development and recurrence are more frequent following DAAs treatment compared to INF -based treatment [157].

Several concerns regarding post-antiviral treatment situation emerge also in the context of the treatment of hepatitis B [158]. In particular, in a retrospective study examining data from large samples, with a clinically and community-wide heterogeneous court of patients with American and Taiwanese chronic hepatitis B, the effects of treatment with peginterferon or oral anti-HBV nucleoside/nucleotide analogue (Nucs) have been evaluated [158]. The study found a significant benefit from treatment both in those at high risk of HCC development such as male gender, older age, cirrhosis, and high REACH-B scores, but also in patients defined as low risk as female gender, younger age, lack of cirrhosis, HBeAg negative, normal to minimally elevated ALT levels, and low-to-middle REACH-B scores (<14) [158].

## 7. Conclusions and Future Perspectives

**Figure 3:**
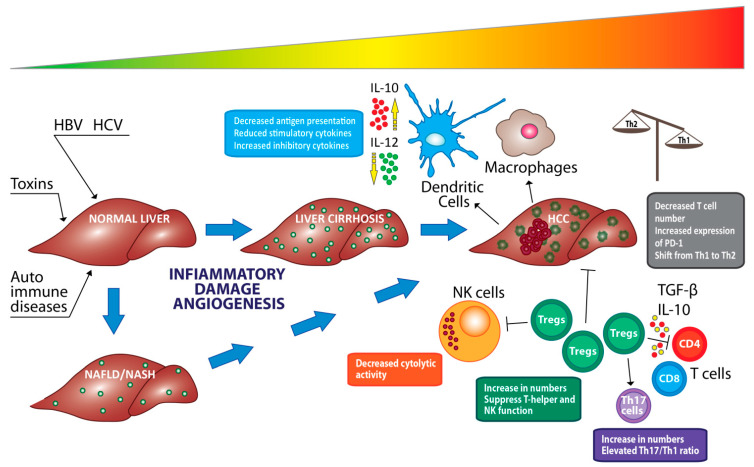
Immune system involvement in the pathogenesis of HCC. Involvement and cross talk between immune cells in the creation of an immunosuppressive environment that allows the tumor to evade immunosurveillance through multiple mechanisms. These mechanisms allow the accumulation of immunosuppressive cell populations resulting in the remodeling of the TME through pathogenic immune factors that induced immuno-imbalance and the development of HCC.

Immune surveillance against cancer exerts a role allowing or preventing cancer cell survival and growth. In particular, immune surveillance can be capable to prevent or fight an effective metastatic process, while on the other hand, the unbalance of immune surveillance processes can induce HCC recurrence. Several factors contribute to the balancing of immune surveillance including HCC patient-specific inflammatory/immune phenotype that, in turn, is influenced by the modulation of inflammation process peculiar of the viral infection as well as by possible modification through effective therapies (Figure 3).

It is possible that the viral infection, being responsible of stromal cell activation as well as of the recruitment of lymphocytes, can be involved in the inhibition of cancer cell growth. In this context, the DAA therapy can quickly reduce the presence of HCV, thus also reducing the number and action of immune system related cells. Generally, this mechanism is associated with a reduction of inflammation signals (i.e., normalization of transaminases). Thus, there is the possibility that tumor clones progress and for this reason they can be recognized as a recurrence in the presence of an abrupt resolution of the chronic inflammatory state (i.e., chronic hepatitis C) capable to abolish the immune ‘‘brake” to tumor progression [152].

Following this possible interpretation, it could be assumed that, although HCV infection is a risk factor for hepatocarcinogenesis, it may somehow be able to sustain patients from relapses of HCC during the period of liver regeneration. During this protumorigenic phase, the absence of the virus could “unmask” pre-existing cancerous lesions supporting their development and progression [139].

A possible explanation for the fact that, in the case of interferon-based HCV therapy, an acceleration of the tumor recurrence or progression does not appear, is that interferon has immunostimulating effects and for this reason could also have antitumor effects, thus reducing tumor progression. On the contrary, DAA agents do not show immunostimulating effects. However, interferon therapies are not generally used in patients with advanced liver disease where there is the higher risk of HCC onset. On the other hand, the relationship between antiviral HBV treatments and risk of developing HCC, or relapse after HCC treatments, has not yet been elucidated.

In conclusion, the clinical appearance of tumors in an accelerate rate following SVR seems to be due to either the lack of immune surveillance or liver regeneration or both; however, this issue is still to be fully clarified. The recent data seem to suggest caution in the administration of DAA therapies, especially in the case of a concomitant administration of specific HCC treatments. In particular, in several cases, one possible option might be to undertake HCV therapies when the HCC tumor has been already adequately treated. Moreover, more frequent follow-up intervals might be applied after DAA therapies.

## Figures and Tables

**Table 1:** Main gene alterations in HCC.

Gene	Pathway	Gene Function Encoded [51]	Alteration in HCC [34]
*TERT*	Telomere maintenance	Telomerase enzyme that keeps the telomere structure stable by adding repeated segments of DNA.	Promoter mutation Amplification
*TP53*	Cell cycle control	The oncosuppressor protein that regulates cell division by preventing cells from proliferating uncontrollably.	Mutation or deletion
*RB1*	Cell cycle control	A tumor suppressor protein that prevent excessive cell growth by inhibiting cell cycle.	Mutation or deletion
*CTNNB1*	WNT pathway	This gene regulates cell growth adhesion between cells and differentiation.	Mutation
*AXIN1*	WNT pathway	Tumor suppressor protein, inhibitor of the Wnt signaling pathway.	Mutation
*ARID1A*	Epigenetic and chromatin remodeling	Member of the SWI/SNF family with helicase and ATPase activities that regulate transcriptional activation and repression of selected genes.	Mutation
*ARID2*	Epigenetic and chromatin remodeling	Tumor suppressor gene with a role in cell lineage gene regulation, cell cycle control, transcriptional regulation, and chromatin structure modification.	Mutation
*NFE2L2, KEAP1*	Oxidative stress	Proteins involved in response to injury and inflammation with production of free radicals.	Mutation
*VEGFA*	Angiogenesis	Induces endothelial cell proliferation, promotes cell migration and invasion, and inhibits apoptosis.	Focal amplification

In the table, the main genetic alterations deregulating key pathways in HCC pathogenesis and proliferation are described.

**Table 2:** Modulation of the TME by immune system-related cells in HCC.

Cell Type	Function in HCC
Tregs	Suppress CD8+ T cells-mediated immunity and pro-inflammatory signals.
	Downregulate molecules involved in T cell activation with damage cytotoxic effects [56,57,59].
MDSCs	Suppress CD8+ T cells function via multiple mechanisms.
	A potent immune suppressive mediator in HCC.
	Poor activation of TIL, inhibition of interferon γ production [62,64,66].
NK	A drastic reduction in the number of peripheral NK cells accompanied by a reduced cytotoxic capacity as well as the production of IFN-γ [67,68,71], with a close correlation to cancer cell apoptosis and patient survival.
Dendritic cells	Immunoregulatory action with the involvement of other lymphocytes [77,78].
CAF	Production of extracellular matrix proteins that support the development of the fibrotic state [86].
	Regulation of neutrophils through the involvement of the IL6-STAT3-PDL1 signaling of pathway and induction of chemotaxis of neutrophils [87,88].

In the table, the main functions of immune system-related cells in the modulation of the HCC TME are summarized.

**Table 3:** Phase 3 interventional studies for HBV treatment terminated with results, drug family, and conditions.

Drug Family	Interventions	Conditions	National Clinical Trial Number
Nucleoside analogue, interferon	Entecavir and peginterferon	Hepatitis B	NCT01369199
Nucleoside analogue, placebo	Drug: telbivudine Drug: placebo	Chronic hepatitis B	NCT02058108
Recombinant interferon alfa-2b is covalently conjugated with monomethoxy polyethylene glycol. The conjugation of PEG (bis-monomethoxy polyethylene glycol) at interferon alpha-2a forms a alpha-2a pegylated interferon (PEGASYS)	Biological: PEG-Intron™ Biological: PEGASYS™	Chronic hepatitis B	NCT01641926
Nucleoside analogue and nucleotides inhibitors of reverse transcriptase Nucleoside analogue and nucleotides inhibitors of reverse transcriptase	Drug: entecavir + tenofovir Drug: adefovir + continuing lamivudine	Chronic hepatitis B	NCT00605384
Synthetic thymidine nucleoside analogue + peginterferon alfa-2a	Drug: Telbivudine (LdT) Drug: peginterferon alpha-2a	Hepatitis B	NCT00412750
Synthetic thymidine nucleoside analogue Nucleoside reverse transcriptase inhibitor (NRTI)	Drug: telbivudine Drug: adefovir dipivoxil	Hepatitis B	NCT00376259
nonpeptidic protease inhibitor (PI) + nonpeptidic protease inhibitor (PI)	Drug: tipranavir Drug: ritonavir	HIV Infections	NCT00447902
Antihemorrhagic	Drug: eltrombopag Drug: placebo	Non-alcoholic steatohepatitis chronic liver disease HCV Nonalcoholic steatohepatitis (NASH) HIV infection Thrombocytopenia Hepatitis C virus	NCT00678587

Summarized are the clinical trials found on ClinicalTrials.gov by searching the keywords “HBV”, “terminated”, “with results”, “interventional” and “phase 3”.

**Table 4:** Phase 3 interventional studies for HCV treatment terminated with results, drug family, and conditions.

Drug Family	Interventions	Conditions	National Clinical Trial Number
NS5A/non-nucleoside polymerase inhibitor (NNPIs) NS5B Nucleoside analogue	Drug: LDV/SOF Drug: RBV	Hepatitis C Virus Infection	NCT02600351
DAAs, nucleoside analogue, the conjugation of PEG (bis-monomethoxy polyethylene glycol) + interferon alpha-2a forms a alpha-2a pegylated interferon (PEGASYS), highly active antiretroviral therapy	Drug: telaprevir Drug: ribavirin, Biological: pegylated interferon Alfa-2a Drug: highly active antiretroviral therapy (HAART)	Hepatitis C	NCT01467479
NS5A inhibitor, NS3/4A inhibitor, non-nucleoside polymerase inhibitor (NNPIs) NS5B	Drug: ombitasvir/paritaprevir/ritonavir and dasabuvir Drug: ombitasvir/paritaprevir/ritonavir Drug: ribavirin	Chronic Hepatitis C Infection	NCT02504099
Cyclophilin inhibitor, peginterferon Alfa-2°, Nucleoside analogue	Drug: alisporivir Drug: peginterferon alfa-2a Drug: ribavirin	Hepatitis C	NCT01500772
Interferon, glycyrrhizin-containing preparation	Biological: peginterferon alfa-2b (PegIFN-2b) Drug: comparator: stronger neo-minophagen C (SNMC)	Hepatitis C, Chronic	NCT00686881
Cyclophilin inhibitor, NS3 inhibitors, peginterferon alfa-2a, nucleoside analogue	Drug: alisporivir Drug: boceprevir Drug: peginterferon alfa-2a Drug: ribavirin	Hepatitis C	NCT01446250
Interferon + nucleoside analogue	Drug: combination of pegylated interferon alfa-2b (PEG) and ribavirin (RBV)	Hepatitis C, Chronic	NCT00423800
Interferon + nucleoside analogue	Biological: PEG-Intron (peginterferon alfa-2b) Drug: rebetol (ribavirin)	Hepatitis C, Chronic	NCT00441584
DAAs, interferon, nucleoside analogue	Drug: telaprevir Drug: pegylated interferon Alfa-2a Drug: ribavirin	Hepatitis C, Chronic	NCT01459913
Interferon, nucleoside analogue, DAAs	Drug: pegylated interferon Alfa 2a Drug: ribavirin Drug: telaprevir	Infection	NCT01821963
Antihemorrhagic	Drug: eltrombopag	Thrombocytopenia Hepatitis C	NCT01821625
NS3 protease inhibitor, NS3 protease inhibitor, interferon, nucleoside analogue, other	Biological: boceprevir Biological: narlaprevir Biological: peginterferon alfa-2b Drug: ribavirin Other: blood/plasma collection	Hepatitis C, Chronic Hepacivirus	NCT00689390
nonpeptidic protease inhibitor (PI), protease inhibitor	Drug: tipranavir Drug: ritonavir	HIV Infections	NCT00447902
Antihemorrhagic	Drug: eltrombopag Drug: placebo	Non-alcoholic Steatohepatitis chronic liver disease HCV NASH HIV infection Thrombocytopenia Hepatitis C virus HBV Human immunodeficiency virus Liver diseases Hepatitis B virus	NCT00678587

Summarized are the clinical trials found on ClinicalTrials.gov by searching the keywords “HCV”, “terminated”, “with results”, “interventional” and “phase 3”.
